# Functional Nano-Coating Materials by Michael Addition and Ring-opening Polymerization: Reactivity, Molecular Architecture and Refractive index

**DOI:** 10.1038/s41598-018-30458-x

**Published:** 2018-08-09

**Authors:** Kishore K. Jena, Saeed M. Alhassan, Atul Tiwari, L. H. Hihara

**Affiliations:** 1 Hawaii Corrosion Laboratory (HCL), Department of Mechanical Engineering, University of Hawaii at Manoa, Honolulu, HI 96822 USA; 2 Department of Chemical Engineering, Khalifa University of Science and Technology (KUST), The Petroleum Institute, PO Box 2533 Abu Dhabi, United Arab Emirates

## Abstract

Understanding the molecular interaction and morphology of organic-inorganic hybrid materials is an important and fundamental assignment to develop novel high-performance materials. In this work, we developed two types of hybrid coating materials by using different silane coupling agents via Michael addition reaction and ring-opening polymerization. The changes in molecular interaction and morphology of the hybrid coatings due to chemical composition and curing temperature were studied by electron microscopy, spectroscopy and solid state ^29^Si nuclear magnetic resonance analysis. Fundamental differences were observed in HYBRID I and HYBRID II coatings during the nucleation stage that was dependent on the curing temperature. Higher curing temperature of the hybrid coatings resulted in improved uniformity and greater crystallinity of dispersed phases, and better control of the morphology compared with coatings cured at lower temperatures. The higher curing temperature provided more consistent nucleation sites for the growth of larger nanostructures of desired characteristics (e.g., size and surface features). There is great flexibility in synthesizingg these hybrid materials where different structure and morphology can be achieved to produce materials whose applications can range from adhesives to protective coatings. Refractive index results revealed that HYBRID I (90 °C) coating showed higher refractive index than HYBRID II (90 °C) coating.

## Introduction

The successful development of hybrid material depends upon the ability to control the material structure to achieve desirable properties^1–4^. The material structure can be controlled by tailoring molecular-size and surface features^5^. The molecular-size is affected by the arrangement of molecules, which determines the final form and shape. Surface features are affected by the chemical and structural characteristics of the surface atoms. The molecular size and surface features govern the properties of the hybrid materials.

Research on organic-inorganic hybrid coatings is a rapidly growing field in materials chemistry. Hybrid materials are frequently prepared by the combination of organic and inorganic molecules by electrostatic interactions or chemical bonding between the two components. Sometimes, this can lead to unpredictable properties. The structures and properties of hybrid materials are functions of the organic and inorganic components and synthesis process. Soft chemistry techniques such as sol-gel processing, intercalation, ion -exchange, and molecular grafting^6–10^ are frequently used for the synthesis of organic-inorganic hybrid materials.

Hybrids are an interesting class of materials that potentially have utility in a multitude of applications because they can be tailored to have combinations of excellent properties such as high mechanical strength, ample flexibility, and variable electrical and thermal conductivities. Due to these properties, interest in three-dimensional hybrid material has significantly grown with extensive research efforts ongoing worldwide. Many protocols have been proposed and developed on the interfacial engineering of organic and inorganic materials to optimize the final properties of the hybrid composite materials^9–13^.

D.K. Chattopadhyay *et al*.^14^ reported on the synthesis of hybrid sol-gel coatings derived from glycidyl carbamate resin, 3-aminopropyltrimethoxy silane (APTMS) and tetraethoxyorthosilicate (TEOS) and studied the thermal and mechanical properties of the hybrid coatings. Marija R. Gizdavic-Nikolaidis *et al*.^15^ studied the spectroscopic characterization of GPTMS/DETA and GPTMS/EDA hybrid polymers with scanning electron microscopy (SEM), Raman spectroscopy, and ^13^C and ^29^Si solid-state nuclear magnetic resonance (NMR) spectroscopies, showing extensive crosslinking within the GPTMS/DETA and GPTMS/EDA hybrids at pH 10.

Schmidt and Krug *et al*.^16^ synthesized cross-linked ormosils from silanes containing epoxide, vinyl, and methacrylate functionalities crosslinked with amines, bisphenols, and imidazoles. T.L. Metroke *et al*.^17^ have investigated primary aliphatic amines (i.e., diethylenetriamine, triethylenetetramine, and tetraethylenepentamine) and super acids (i.e., CF_3_SO_3_H and HPF_6_) as room-temperature curing agents for hybrid organic–inorganic thin films. M.S. Donley *et al*.^18^ reported on key parameters in the beginning stage (sol–gel processing), coating application and the end stage (curing) processes in the GPTMS/TMOS system. They characterized the solution chemistry of the hybrid materials by NMR, light scattering and GPC techniques. They used solid state NMR, XRD, AFM and grazing angle XPS for the analysis of chemical composition, structure, morphology and the surface chemistry of the SNAP coatings. The studies above just describe a small example of the possibilities of hybrid materials that remain largely unexplored, but where endless new types may be synthesized and unique properties achieved.

In this work, we focused our synthesis of hybrid coatings on Michael addition reaction and ring opening polymerization techniques. The possibilities to use basic synthesis via Michael addition and ring opening polymerization to obtain hybrid materials have not been well explored. We focused on the elucidation of the structure and on the organization of hybrid building blocks. Using Michael addition reaction and ring opening polymerization, we prepared two high cross-linked hierarchically assembled hybrid films on an aluminum alloy surface (Scheme SI-1). The changes in molecular structure and morphology of the hybrid materials with respect to curing temperature were characterized. The structures of the hybrid coatings on the aluminum alloy were characterized by FTIR, Raman and solid state ^29^Si NMR. The surface morphology of the hybrid films was studied by TEM, SEM and AFM.

## Experimental

### Materials

(3-Trimethoxysilylpropyl) diethylenetriamine (Mw = 265.43 gmol^−1^), (3-Aminopropyl) trimethoxysilane (Mw = 179.29 gmol^−1^), 3-(Trimethoxysilyl) propyl methacrylate (Mw = 248.35 g mol^−1^) and Boron trifluoride diethyl etherate (BF_3_·O(C_2_H_5_)_2_ (Mw = 141.93 g mol^−1^) were obtained from Aldrich Co., USA. (γ-Glycidyloxypropyl) trimethoxysilane was purchased from Alfa Aesar Co., USA. All chemicals were used as received without further purification.

### Synthesis of organic-inorganic hybrid sols

The synthesis rout of the organic-inorganic hybrid sols is schematically shown in Scheme SI-1. The hybrid materials were prepared by two novel mechanisms which were Michael addition and ring-opening polymerization. The synthesis of the hybrid materials was achieved by using commercially-available sol-gel precursors as the starting material. Hydrolysis and condensation reactions of these precursors with water in a one-to-one (1:1) OMe–H_2_O mole ratio produced the corresponding hybrid in high yields, as evident by the presence of the FTIR and Raman Si-O-Si peaks.

#### Preparation of hybrid sol by Michael addition reaction

3-trimethoxysilyl propyl methacrylate (*TMSPM*) (21.2 g) was poured into a 250 ml three-neck flask that was fitted with a condenser, a drop funnel and a N_2_ inlet. 3-trimethoxy silyl propyl diethylene triamine (TSPDT) (4.8 g, 0.018 mol) and 3-amino propyl trimethoxy silane (APTMS) (1.2 g, 0.006 mol) were mixed in 20 g of ethanol (CH_3_CH_2_OH; Alfa Aesar) and then introduced slowly into the flask at 30 °C. After addition of the amine precursors the reaction mixture was vigorously stirred under N_2_ at 50 °C for 24 h resulting in a pale yellow hybrid solution. Hydrolysis of the hybrid solution was performed at a 1:1 mole ratio of H_2_O:Si–OMe. Boron trifluoride etherate BF_3_O (Et)_2_ (0.05 wt % of the total mixture) was used as the catalyst to promote the reaction. The developed hybrid sol will be referred to as HYBRID I.

#### Preparation of hybrid sol by epoxy rings opening mechanism

γ-glycidoxypropyltrimetoxysilane (GLYMO) (20.12 g) was poured into a 250 ml three-neck flask that was fitted with a condenser, a drop funnel and a N_2_ inlet. 3-trimethoxy silyl propyl diethylene triamine (TSPDT) (4.8 g, 0.018 mol) and 3-amino propyl trimethoxy silane (APTMS) (1.2 g, 0.006 mol) were mixed in 20 g of (CH_3_CH_2_OH; Alfa Aesar) and then introduced slowly into the flask at 30 °C. After addition of the monomers, the hybrid solution was stirred vigorously at 50 °C under N_2_ environment for 24 hours to complete the reaction. The 1:4 molar ratio of TSPDT: GPTMS and 1:2 molar ratio of APTES: GPTMS were chosen to ensure that there was a 1:1 molar ratio of GPTMS to the active H atoms (in NH_2_ and NH groups), such that one molecule of GPTMS was present for each active hydrogen in the amino groups of the amine precursors. Boron trifluoride etherate BF_3_O (Et)_2_ (0.05 wt % of the entire mixture) was used as the catalyst to enhance the opening of the epoxy rings of GPTMS by the amino groups and also the sol-gel reaction. Hydrolysis was performed at the 1:1 ratio of H_2_O:Si–OMe. The developed hybrid sol was referred to HYBRID II. Detail of substrate preparation and film deposition is reported in the supporting information.

## Results and Discussion

### Raman analysis

Raman spectroscopy was used to characterize the molecular bonding, which showed up in spectral band energy and intensity, and also allowed the *in situ* examination of the intermolecular interactions. The analysis gave some insight into the 3-dimensional arrangement and interactions of different functional groups on the observed bulk properties.

**Figure 1:**
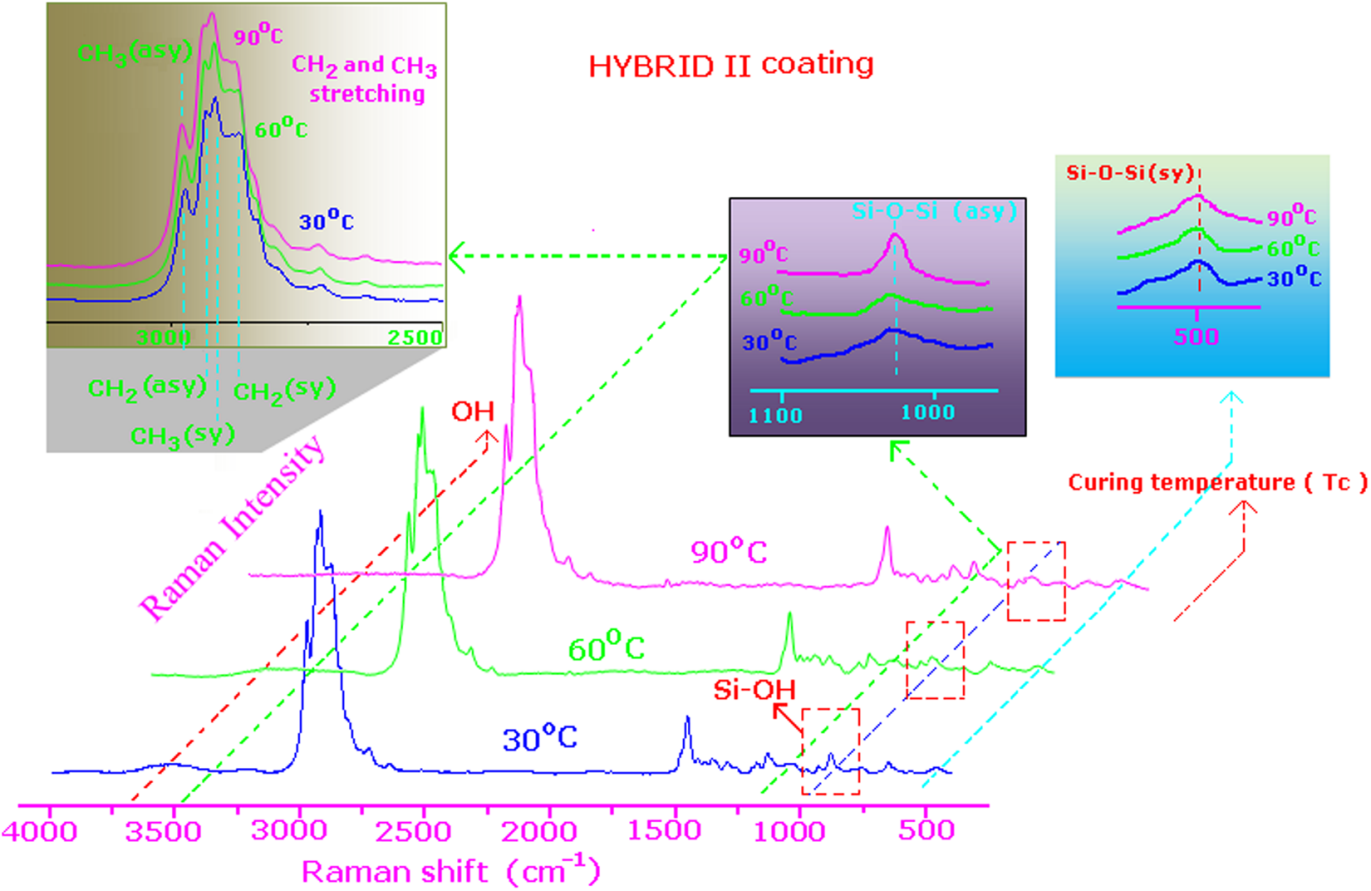
Full Raman Spectra of HYBRID II at different curing temperature (30, 60, and 90 °C).

**Figure 2:**
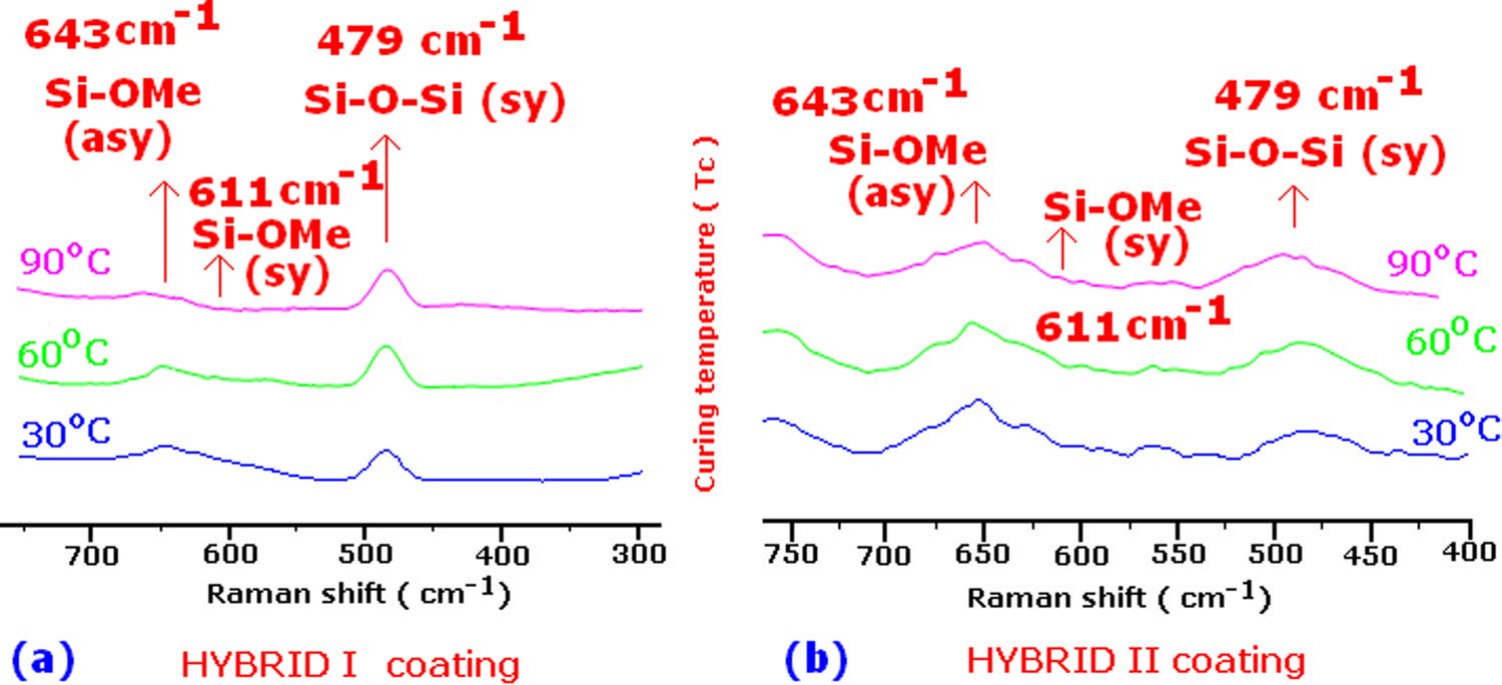
The expanded zone of HYBRID I (**a**) and HYBRID II (**b**) in the spectral range from 700 to 300 and from 750 to 400 cm^−1^. This figure identified the difference in intensities of both hybrid coatings. This was slight disappearance of the methoxy groups with increasing temperatures. The details are described in discussion.

Figure SI-1 and Fig. 1 shows the Raman spectra of HYBRID I and HYBRID II after different curing temperatures in the spectral range 4000–300 cm^−1^. The expanded zone of the HYBRID I and HYBRID II spectra in the spectral range from 700 to 300 and from 750 to 400 cm^−1^ is shown in Fig. 2(a,b), respectively. The spectra of HYBRID I and HYBRID II exhibited a reduction of the symmetric ν_s_(Si–OMe) and asymmetric ν_as_(Si–OMe) bands at 611 and 643 cm^−1^ with increasing temperature. This could be due to several reasons: There was the beneficial effect of the 1:1 molar ratio of H_2_O: Si–OMe present in the sol, allowing the -OMe groups to be hydrolyzed. The higher temperatures caused the unreacted –OMe groups to take part in the condensation reaction. The symmetric ν(CH_3_) stretching band at 2843 cm^−1^ intensity slightly decreased with increasing the temperature in both the hybrid system (insert in Fig. SI-1 and 1). This indicated the condensation of the methoxy groups of the sol-gel precursors. The comparison of HYBRID I and HYBRID II coatings shows that the HYBRID II coatings has more unreacted methoxy (Si-OMe) group compared to HYBRID I (shown in Fig. 2(a,b)). This could be due to unsymmetrical nature of the HYBRID II molecules. These figures indicated that the Si-O-Si band intensity increased with increasing temperature in both hybrids and also the Si-O-Si band intensity was higher in HYBRID I compared to HYBRID II. The decreasing of intensity of silanol ν(Si–OH) band at 980 cm^−1^ (Fig. 1) provided the evidence of condensation reactions. The Si–O–Si linkages that formed during condensation were observed as broad bands at 479 and 1078 cm^−1^ ^19,20^.

The spectra in Fig. 2(a,b) and insert in Figs SI-1 and 2 indicated the formation of siloxane network (linear, cyclic and branched) in the hybrid coatings. The siloxane network forms the backbone of the ceramer coatings. Theoretically, we expected that the network could be arranged into three possible structures: the linear, cyclic and branched molecular structures are shown in Scheme SI-3. Both HYBRID I and HYBRID II coatings formed a siloxane network (linear, cyclic and branched) after condensation reaction. Linear, cyclic and branched molecular structures are due to the different magnitude of siloxane network. These three molecular arrangements have very similar wave numbers, making it difficult to de-convolute the Raman Spectra.

The Raman spectra of Hybrid I and Hybrid II displayed the characteristic absorption peaks at 2843 cm^−1^ (symmetric ν(CH_3_) stretching band), 980 cm^−1^ (ν(Si–OH) band), 643 and 611 cm^−1^ (symmetric ν_s_ (Si–OMe) and asymmetric ν_as_ (Si–OMe) bands) and at lower wave number broad band at 479 cm^−1^ (Si–O–Si linkages). The spectra showed (Fig. SI-1 and 1: Expanded zone of Si-O-Si symmetric stretching zone) that the intensity of Si–O–Si bands at 479 and 1078 cm^−1^ increased in the case of HYBRID I compared to HYBRID II, and more siloxane network formed in the hybrid system with increasing temperature. To better characterize the distribution of the molecular network, FTIR analyses were conducted in this work.

### FTIR analysis

Infrared reflectance spectroscopy was also applied to the hybrid coatings cured at the different temperatures. The absorption spectra of cured HYBRID I and HYBRID II (Fig. SI-2) showed absorption maxima based on different vibrations: (i) stretching vibrations of Si-O-Si^21–23^ and Si-O-M (M = A1) bridges between 950–1200 cm^−1^; and (ii) bending vibrations of OH groups between 800–950 cm^−1^. After heating the sample, primary changes occurred between 950–1200 cm^−1^ (Fig. SI-2 (expanded zone)) where the band at 1052 cm^−1^ showed significant attenuation, and the emergence of a new band at 1071 cm^−1^ developed the highest intensity with increasing curing temperature. In addition, the intensity of the band at 1121 cm^−1^ increased and the frequency shifted to 1128 cm^−1^. This implied the formation of more Si-O-Si network in the hybrid. In frequency region 800–950 cm^−1^, the band at 928 cm^−1^ disappeared and was replaced by a broad band centered at 946 cm^−1^. Below 600 cm^−1^, the hybrid coatings were less sensitive to heat treatment and no significant changes to the spectra were observed. Similar behavior was also observed by M. A. Karakassides, D. Gournis and D. Petridis^22^ upon heat treating montmorillonites.

**Figure 3:**
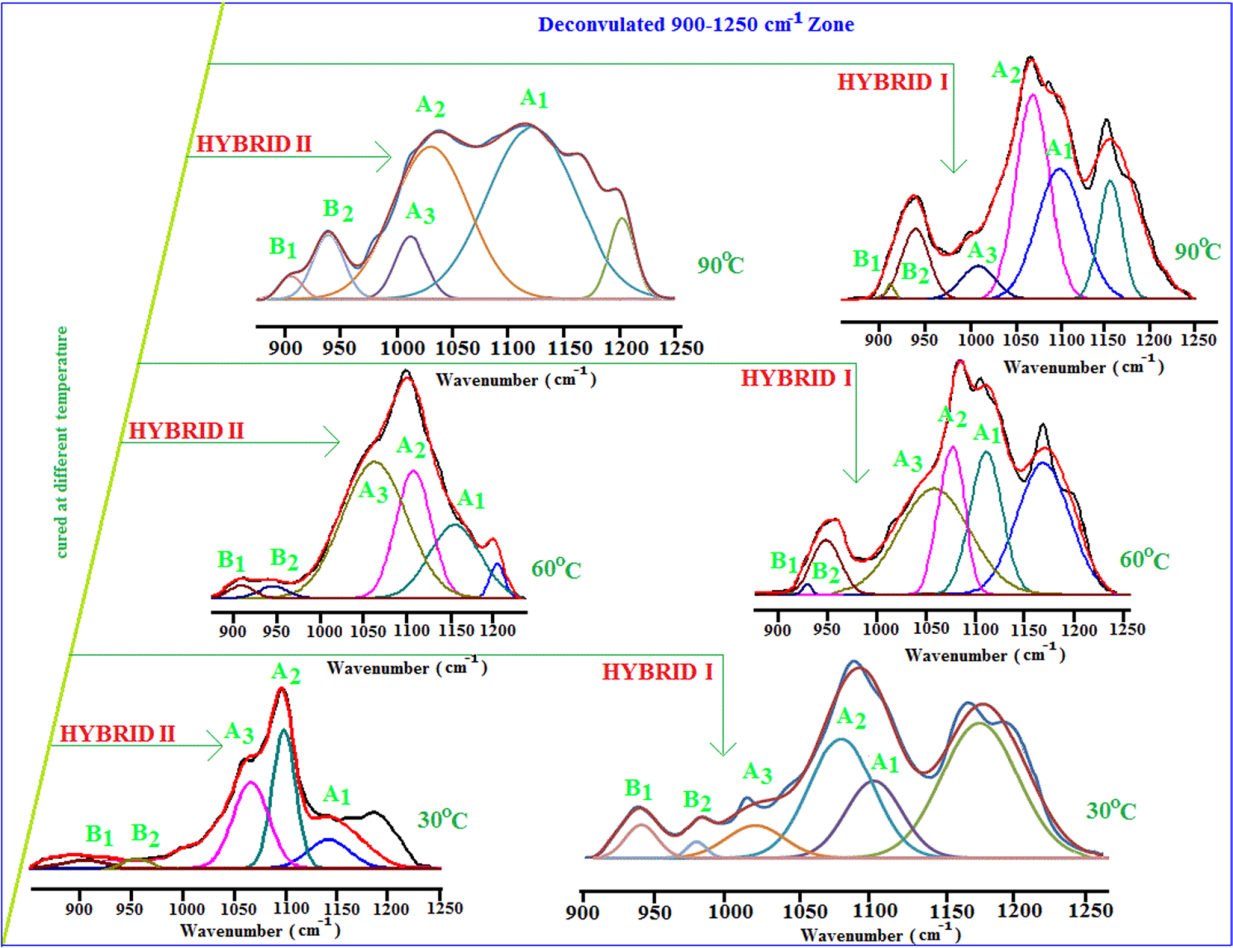
Deconvolution of 900–1250 cm^−1^ zone of hybrid samples.

By the knowledge of the peak positions, the architecture of the inorganic domains can be assessed by applying a deconvolution method of multi-peaks, as shown in Fig. 3. The Origin program was used to perform the peak deconvolution of hybrid coating materials. Deconvoluted spectra of heat-cured HYBRID I and HYBRID II in region of 900–1250 cm^−1^ highlight that the architecture of the inorganic domains in terms of linear, cyclic and branched siloxane arrangements changes with the curing temperature. The deconvolution results from region 900–1250 cm^−1^ agree with previous IR work^21,22,24^ on the Si-O vibrational pattern of organosilicon. The band at 1145 cm^−1^ (A_1_), 1110 cm^−1^ (A_2_) and 1026 (A_3_) cm^−1^ likely arised from the 3D Si–O-C cage structure, network structure and long-chain linear structure of the siloxane, respectively. The results can be explained by considering the growth of the inorganic siloxane networks. The above mentioned three types of structures were shown in the Raman analyses (Scheme SI-3). In the deconvoluted spectra of the 60 °C curing (Fig. 3), the intensity of the component band A_2_ substantially increased; whereas, those near to the band at 1026 cm^−1^ (A_3_) were attenuated. The cage structured band (A_1_) at 1145 cm^−1^ also showed an increase in intensity at 60 °C curing temperature in both the HYBRID I and HYBRID II. At the 90 °C curing temperature, the intensity of the cage-structured band (A_1_) at 1145 cm^−1^ increased significantly, and those of bands A_2_ and A_3_ decreased. The overall studies state that the higher curing temperature allowed the siloxane network to form more rapidly. In region 900–950 cm^−1^, the deconvoluted spectrum of the cured hybrid was fitted with two components bands B_1_ and B_2_. The bands at 928 cm^−1^ (B_1_) and 956 cm^−1^ (B_2_) arised from unreacted OH groups and Si-O-M (metal) bridges in both the HYBRID I and HYBRID II coatings. The FTIR spectra showed that the heat treatment of the coatings decreased in the intensity of the 928 cm^−1^ band and formed a new band (band B_2_) at higher frequency (946 cm^−1^), which were likely attributed to the perturbation of OH vibrations resulting from the heat treatment. In both the HYBRID I and HYBRID II deconvoluted figures; we observed the peak intensity of band B_2_ increased and band B_1_ decreased with increasing curing temperatures. This behavior could be due to the formation of Si-O-M bridges.

It was interesting to compare the Si-O vibrations of the HYBRID I and HYBRID II coatings. Figure SI-2 (expanded region) shows the IR spectra of HYBRID I and HYBRID II for different heat treatments. All of the spectra were normalized for the intensity at the 475 cm^−1^ band, which did not show significant change with heating (expanded zone). Below 600 cm^−1^, no significant differences were apparent. In the region between 900–1250 cm^−l^, changes in the spectra were more pronounced in the HYBRID I sample (expanded zone). The deconvoluted profiles (Fig. 3) showed a large relative increase in the peak of component A_1_ for HYBRID I. In comparison, the band A_2_ and A_3_ in HYBRID I highly decreased in intensity compared to those in HYBRID II, and the bands below 600 cm^−1^ remained nearly constant.

### Solid-state ^29^Si NMR

Solid state ^29^Si NMR spectroscopy was used to characterize the chemical structure of the inorganic network for the hybrids cured at 30, 60 and 90 °C. During condensation reactions, the silicon neighbors changed (i.e., Si-OH to Si–O–Si). The molecular arrangements of the siloxane network in the HYBRID I and HYBRID II materials were analyzed by solid-state ^29^Si NMR, as shown in Fig. SI-3(a,d), respectively. The terminal structural units of siloxane precursors gave rise to signals T^1^, T^2^ and T^3^ that were due to the three functional groups that induced the condensation reactions. The units associated with T^1^ had two residual hydroxyl groups [R-Si (OH)_2_− O−], those associated with T^2^ had one residual hydroxyl group [R-Si (OH)−(O)_2_−], and those associated with T^3^ had all three hydroxyl groups [R-Si (O)_3_−] that took part in the condensation reaction^25^. The T species correspond to the silane hydrolysed groups as shown in Scheme SI-2. Figure SI-3(b,e) shows three peak groups assigned as T^1^, T^2^, and T^3^, respectively, for −49.6 and −49.1 ppm (T^1^), −58.7 and −58.4 ppm (T^2^), and −68.9 and −68.3 ppm (T^3^) in the solid state ^29^Si NMR of both hybrid materials. This suggested that both the hybrid materials had all three structural units. The positions of the T^1^, T^2^ and T^3^ peaks were similar to those reported in literature^26,27^. The splitting of the T^2^ peak shown in Fig. SI-3(b,e) was likely due to the spin–spin splitting of alkyl group. There were less T^1^ units in HYBRID I and HYBRID II materials after 90 °C curing compare to 30 °C curing (Table SI-1). This indicated that some of the T^1^ units transformed into T^2^ or T^3^ units. Figure SI(b,e) show the significant difference in the chemical structure of the hybrid materials. In the HYBRID I, the amount of T^2^ units significantly decreased, and that of T^3^ units increased. This suggested that in HYBRID I, a substantial amount of the T^2^ units reacted in condensation, leading to a larger increase T^3^ units in comparison to HYBRID II. The proportion of the T^1^, T^2^, T^3^ and D_c_ in the hybrid materials was quantitatively determined based on the peak areas of T^1^, T^2^ and T^3^ species. The chemical shift, relative proportions of T^1^, T^2^ and T^3^, and the degree of condensation (D_c_) of the hybrid materials are listed in Table SI-1. The D_c_ of the hybrid materials was calculated from the proportions of T^1^, T^2^ and T^3^ based on the following equation: D_c_ (%) = [T^1^ + 2T^2^ + 3T^3^/3] × 100^28^. The proportion of T^1^ and T^2^ species decreased and T^3^ specie increased with the increasing curing temperature in both (HYBRID I and HYBRID II) hybrids. The D_c_ result shows that the HYBRID I materials underwent slightly higher condensation. The D_c_ depends on the Si–OH condensation in the hybrid materials.

### FE-SEM analysis

**Figure 4:**
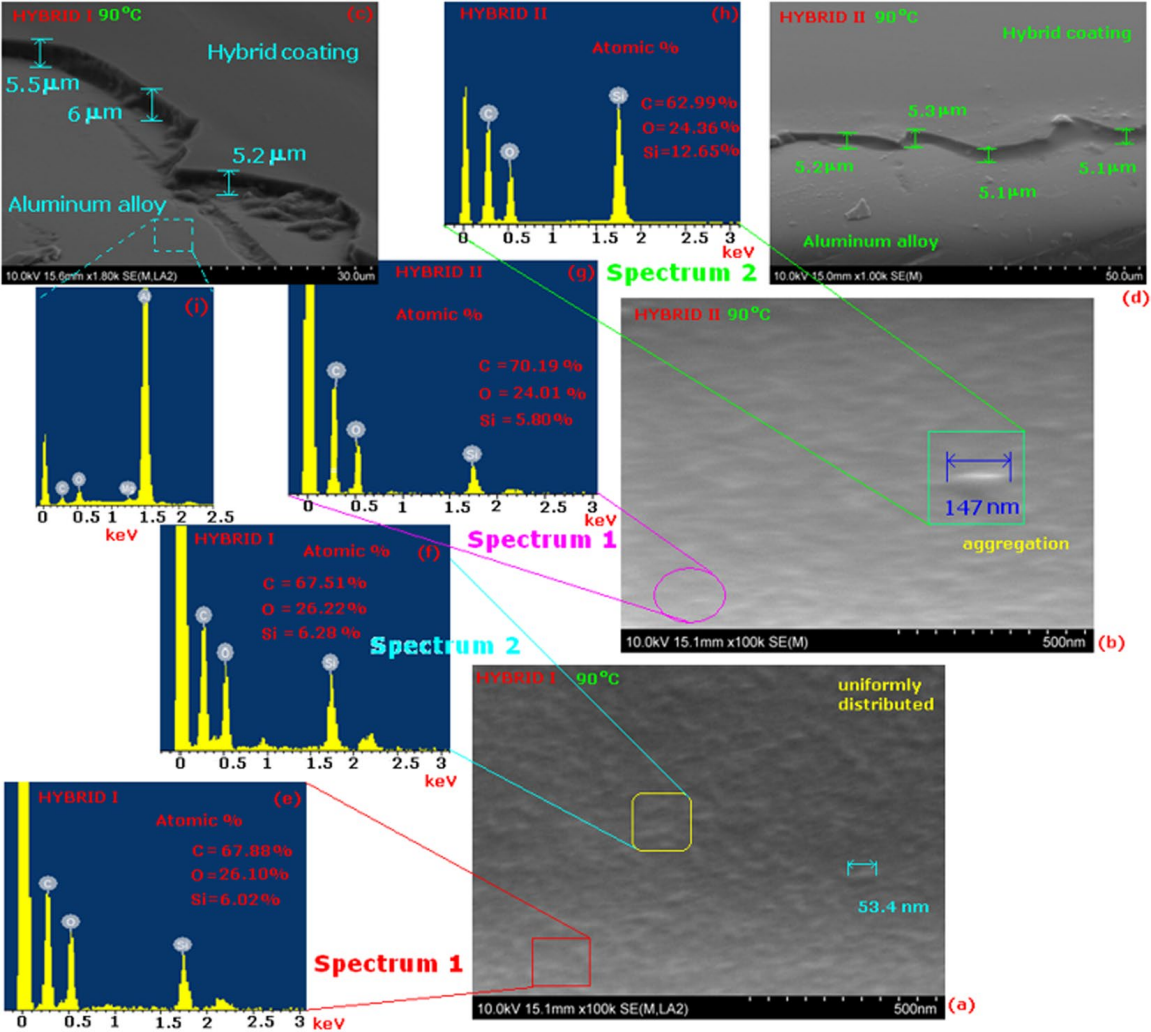
FE-SEM micrographs and EDX spectra of hybrid coatings at 90 °C. SEM images of cross-sectional area of hybrid samples.

A field emission gun-scanning electron microscope (FE-SEM) (Fig. 4(a,b)) was used to investigate the compatibility between the organic and the inorganic phases. The compatibility between the organic and the inorganic phases strongly affects the properties of the hybrid materials^29^. FE-SEM in concert with EDAX was used to investigate the distribution of inorganic phase in the organic-inorganic hybrid matrix. Figure 4(a,b,e–h) present SEM images and EDAX curves of HYBRID I and HYBRID II coatings, respectively. In HYBRID I, the silica particles were less than 100 nm and uniformly dispersed throughout the organic matrix. In HYBRID II, the silica particles were also uniformly dispersed throughout the organic matrix, but in some regions, the particles were aggregated with sizes exceeding 100 nm (e.g., 147 nm particles shown in inset Fig. 4(b)). These results revealed that the HYBRID I coating exhibit better distribution and miscibility between organic and inorganic phases compared to the HYBRID II coating. Figure 4(c,d) show the cross-section FE-SEM micrographs of hybrid films deposited on the aluminum substrate by dip coating. The films were continuous and compact with a thickness of approximately 5–6 μm. The dip-coating process lead to a topographically homogeneous film (Fig. 4(c,d)).

### TEM analysis

Coating structural investigations were performed using TEM. In this work, the HYBRID I and HYBRID II coatings were diluted to 2 wt.% solid content to deposit a thin film on the surface of the copper grid. The hybrid coatings needed to be sufficiently thinned to enable the transmission of the electron beam through the material to generate the structural image. It was anticipated that the general structure observed in the TEM images would be similar to the actual coatings, with the exception that the silica clusters in the actual coatings would be much more closely spaced than observed in the TEM image. In addition, the thicknesses of the actual coatings on the metal substrates were approximately 5–6 μm (as determined by FE-SEM (Fig. 4(c,d)); whereas, the coating thickness on the copper grid surface may be less than 1 μm. Therefore, it is expected that the silica clusters would be overlapping in the 5–6 µm thick coatings.

**Figure 5:**
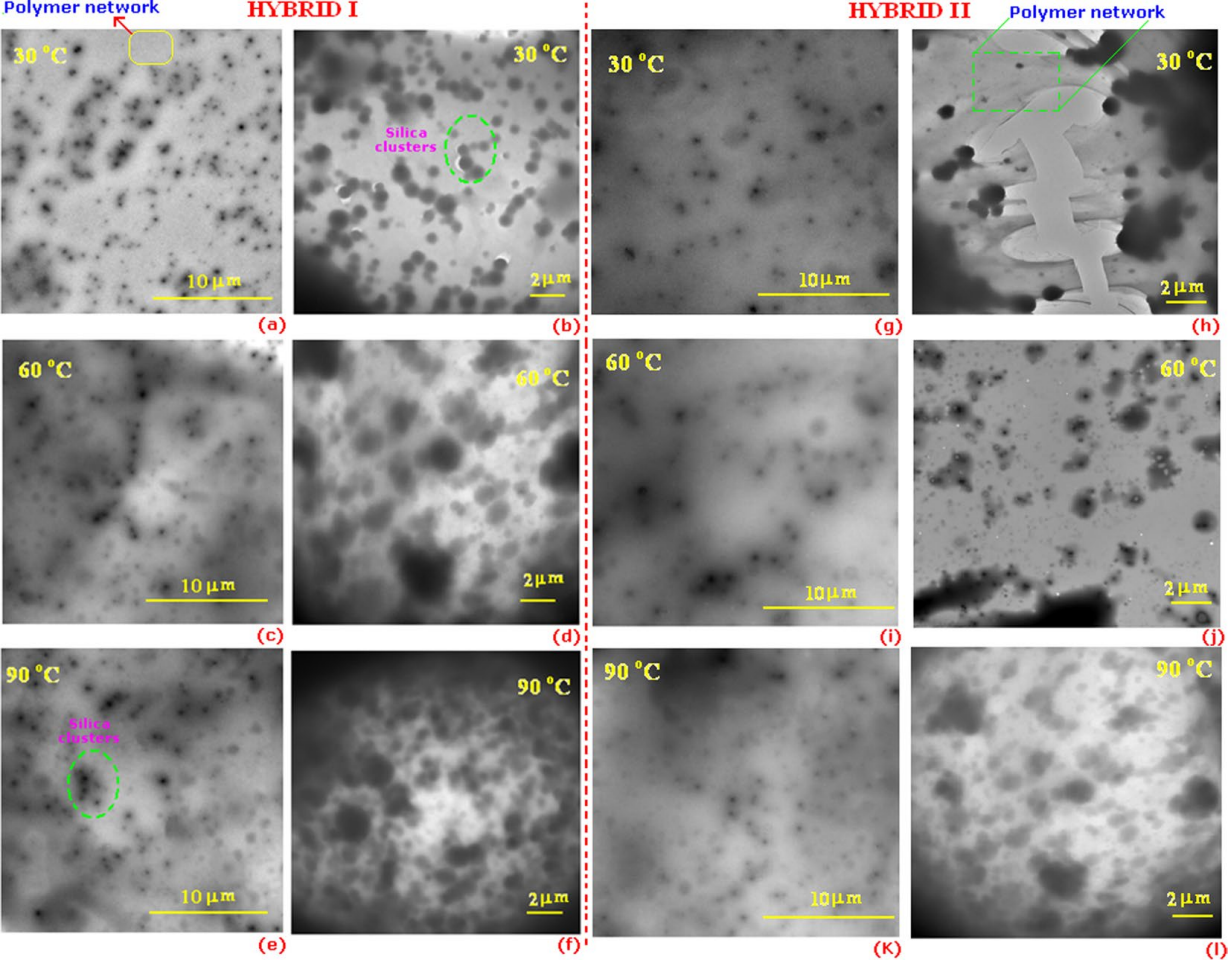
Hybrids characterization by Transmission Electron Microscopy. Transmission electron microscope images of hybrid samples at different curing temperature: 30, 60, and 90 °C.

**Figure 6:**
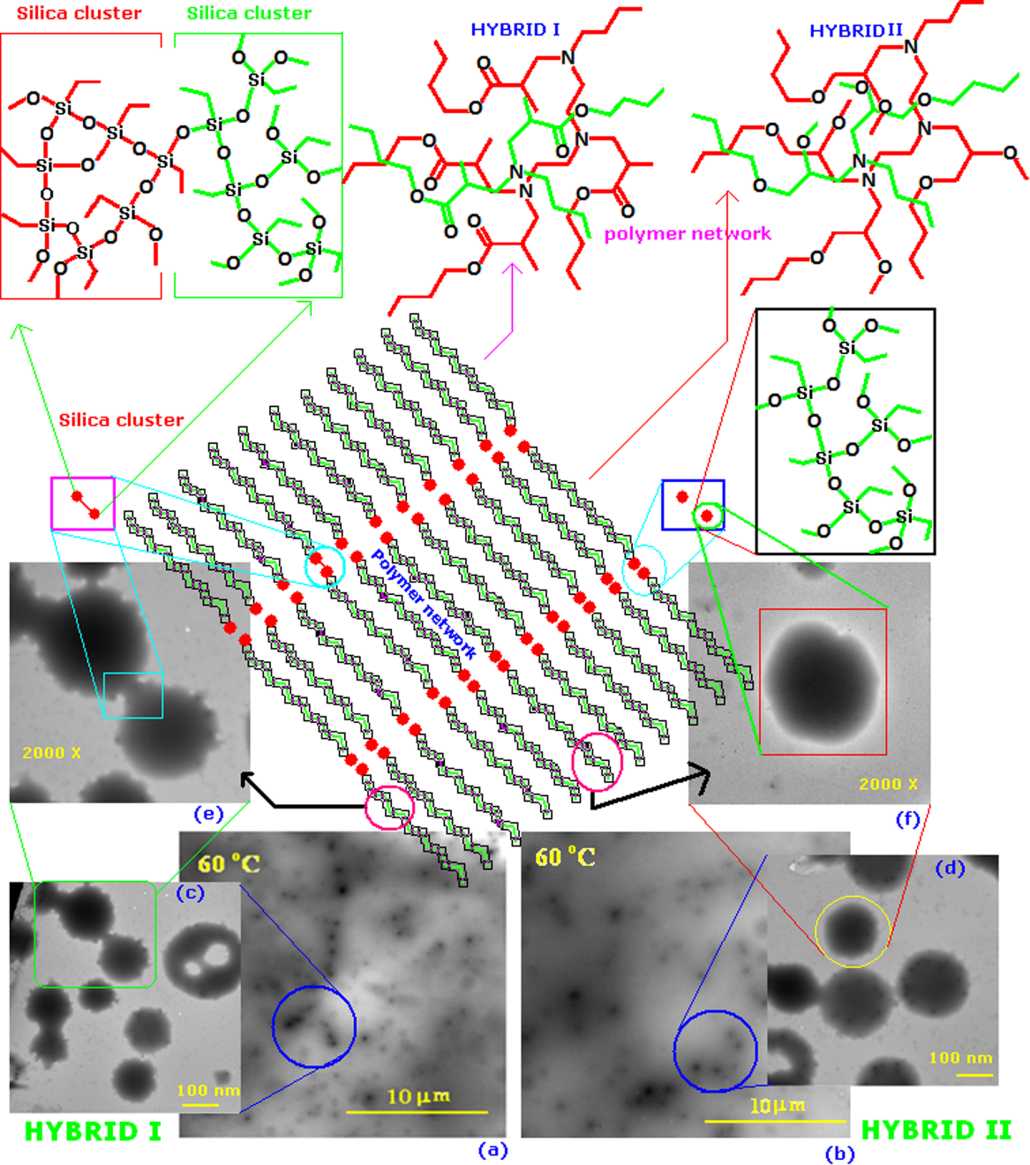
Shows the structural difference between HYBRID I and HYBRID II and structure of silica cluster and polymer network. Silica cluster looks like dark and polymer network grey in color in TEM images. High magnification TEM micrograph of silica particles, illustrating particle links (**c** and **e**).

TEM images of the HYBRID I and HYBRID II coatings cured at different temperatures are shown in Fig. 5(a–l). Both the coatings show silica nano-clusters (dark spots) surrounded by amorphous polymer material (gray region)^30,31^. Note that in the actual coatings, the silica clusters should be much more densely packed. Slightly different microstructures were obtained when the coatings were cured at different temperatures (i.e., 30 °C, 60 °C and 90 °C). The silica clusters appear to be less distinct, and sometimes grew in size as the curing temperature increased. It is possible that smaller silica clusters were consumed by larger clusters as the inter-connectivity of the silica network increased. The free silanol groups in the silica domains condensed with each other by forming Si-O-Si linkages. These linkages brought the molecules closer together causing the silica domains or clusters to grow. The size of the silica particle in the hybrid films was estimated from the TEM images. The structure of silica clusters and polymer materials are shown in Fig. 6.

To further study the silica clusters, TEM images of only the dark cluster regions were investigated for coatings cured at 60 °C. The dark clustered regions (in Fig. 6(a–f)) were comprised of individual silica particles within a polymer network (Fig. 6 (model structure)). Interaction between the individual silica particles was observed for both coatings; however, the extent of the interaction appeared to be greater for the HYBRID I coating. The surface of the HYBRID I particles (Fig. 6(c)) appear to have more “fingers” that those of HYBRID II. This could be observed due to the symmetrical nature of HYBRID I. HYBRID I was synthesized by the Michael addition reaction where the hybrid molecules are more symmetrical in nature compared those in the HYBRID II coating, which was prepared by a ring opening mechanism. In the case of the HYBRID I coating, large Si-O-Si groups can be formed since the siloxane clusters are arranged uniformly. However, in the HYBRID II coating, Si-O-Si and Si-O-C networks are formed, which may impede the formation of continuous silica networks.

### AFM analysis of hybrid coatings

**Figure 7:**
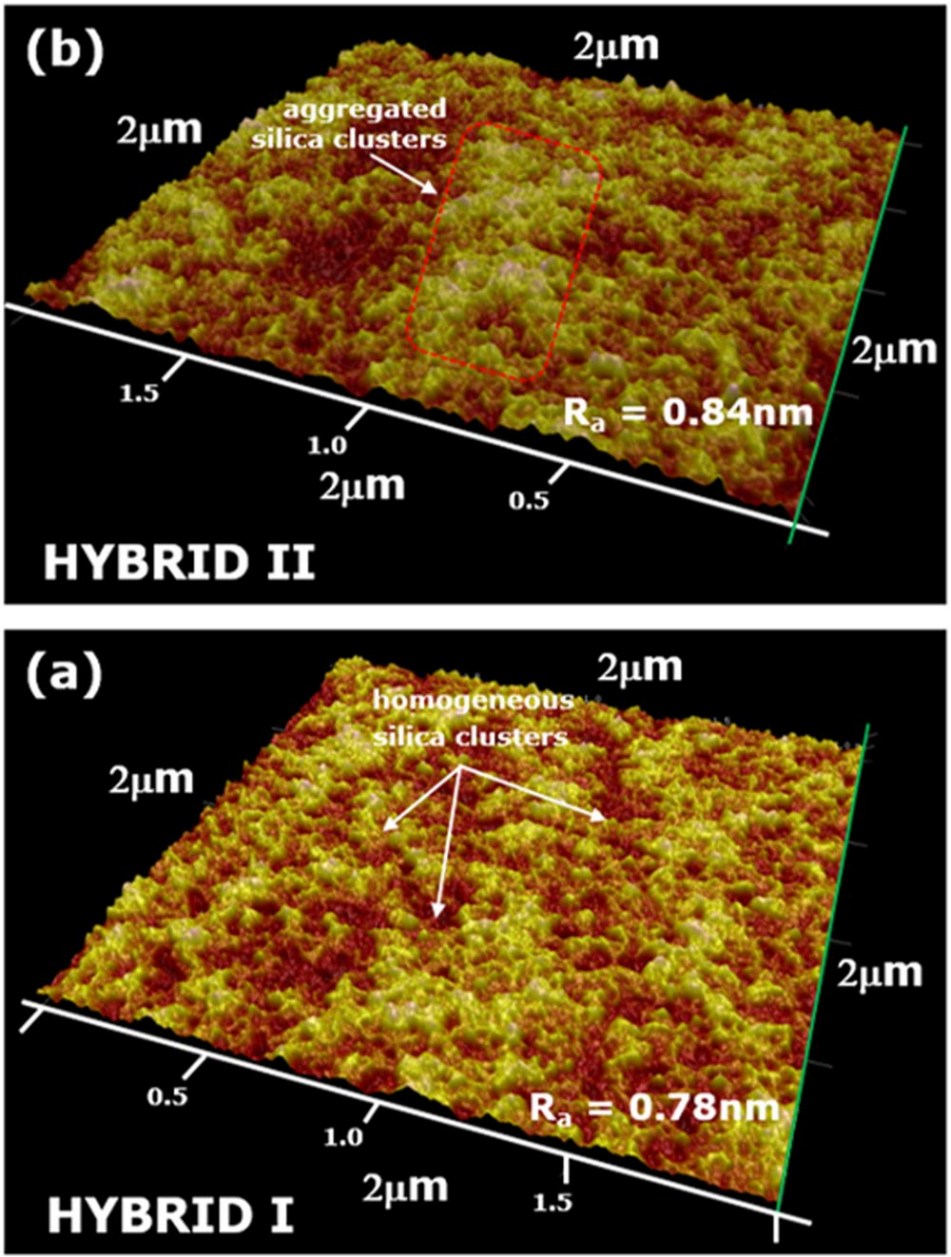
AFM images of the surface of hybrid coatings formulated by using (**a**) Michael addition reaction (HYBRID I); (**b**) Rings opening mechanism (HYBRID II) at 90 °C curing.

Hybrid coatings surface morphology after 90 °C curing was investigated by the AFM analysis. In this study, a comparison was made between HYBRID I hybrid coating prepared by Michael addition reaction and HYBRID II coatings prepared by Rings opening mechanism. The mirror polished aluminum surface was prepared to coat the hybrid coating to reduce the surface roughness and form thinner and uniform coatings. Surface morphology and average surface roughness (*R*a) of the resulting hybrid coatings are compared in Fig. 7(a,b). The hybrid coating prepared by Michael addition reaction (Fig. 7(a)), the surface morphology was uniform with an absence of any defects, providing *R*a of about 0.78 nm but HYBRID II hybrid coating shows small defects with aggregation in the coating morphology (Fig. 7(b)), providing *R*a of about 0.84 nm.

### Refractive index study of hybrid coatings

The refractive index of HYBRID I and HYBRDI II coatings were measured by variable angle spectroscopy Ellipsometer (VASE −32, J.A. Woollam Co. Inc) in the wavelength range from 400 to 1700 nm and angle of incidence from 40 to 85°. The HYBRID I and HYBRDI II coatings were spin-coated on an optical glass plate. The Refractive indices (RI) values as a function of wavelength for HYBRID I (90 °C) and HYBRID II (90 °C) coating films observed at 550 nm were 1.623 and 1.597, respectively. It was confirmed that, the HYBRID I coating exhibited slightly higher RI values compared to that of the HYBRID II coating. This could be due to increased connectivity of the siloxane network and increased condensation reaction rate of the HYBRID I coating. The relatively similar refractive indices in the HYBRID I and HYBRID II coatings could be due to the refractive index of the organic components. The findings were very promising as compared with TiO_2_, ZnS, SiO_2_ and PbS based coatings, such as, TiO_2_-PGMA (60wt %) (1.77)^32^, PVAL-TiO_2_ (1.6)^33^, PbS-PTU (1.762, 632.8 nm)^34^, ZnS-PTU (1.754,632.8 nm)^35^ and MPS-SiO_2_ (1.420)^36^, etc. Hybrid coatings that have high refractive index may have utility in advanced optoelectronic applications. In this work, we have designed hybrid coatings from siloxane network without using TiO_2_, PbS and ZnS nanoparticles and have found promising results.

## Conclusions

The inherent advantages of sol-gel processing with the ability to chemically modify the sol-gel precursor allowed tailoring of specific material properties. The aim of this study was to provide structural and morphological information of hierarchical-structured hybrid coatings prepared from silane-functionalized precursors. In this work, hybrid coatings were synthesized by using Michael addition reaction and ring opening polymerization techniques. In the Michael addition reaction, the hybrid coatings were formed more uniformly than ring-opening polymerization reaction due to the chemical structure of 3-trimethoxysilyl propyl methacrylate (*TMSPM*). FTIR and Raman study showed that the Si–O–Si bands at 479 and 1078 cm^−1^ increased in case of HYBRID I compared to HYBRID II, and more siloxane network formed in the hybrid system with increasing temperature. According to SEM microscopy, the coatings were dense, smooth and without distinguishable defects. The thickness of prepared hybrid coatings was in the range from 5–8 μm. The presence of silica clusters in the hybrid film was supported by TEM and AFM studies, which indicated that larger nanoparticles were formed by the coalescence of smaller nanoparticles. These results showed that hierarchical structured hybrid materials prepared by Michael addition reaction exhibited better structural arrangement, smoother coating surface and a better homogeneous distribution of silica domains in the hybrid system compared to hybrid materials prepared by ring opening polymerization. Promising high refractive indexes were also achieved.

## Electronic supplementary material

Supplementary Information

## References

[1] Zang L, Chen D, Cai Z, Peng J, Zhu M (2018). Preparation and damping properties of an organic–inorganic hybrid material based on nitrile rubber. Composites Part B: Engineering.

[2] Li C (2017). Silver nanoparticles/polydimethylsiloxane hybrid materials and their optical limiting property. Journal of Luminescence.

[3] Zhao H (2018). Development of solution processible organic-inorganic hybrid materials with core-shell framework for humidity monitoring. Sensors and Actuators B: Chemical.

[4] Toshima N (2017). Recent progress of organic and hybrid thermoelectric materials. Synthetic Metals.

[5] Desautels R (2012). Increased surface spin stability in γ-Fe2O3 nanoparticles with a Cu shell. Journal of Physics: Condensed Matter.

[6] Blas F (2018). Processing thermal barrier coatings via sol-gel route: Crack network control and durability. Surface and Coatings Technology.

[7] Raimondo M (2017). Superhydrophobic properties induced by sol-gel routes on copper surfaces. Applied Surface Science.

[8] Allauddin S, Narayan R, Raju K (2013). Synthesis and properties of alkoxysilane castor oil and their polyurethane/urea–silica hybrid coating films. ACS Sustainable Chemistry & Engineering.

[9] Gao Y (2001). Organic− Inorganic Hybrid from Ionomer via Sol− Gel Reaction. Chemistry of materials.

[10] Kikuchi S, Saeki T, Tabata K, Ohta K (2009). Study on the gel aging of nano silica sol produced by a Y-shaped reactor. e-Journal of Surface Science and Nanotechnology.

[11] Kaide A, Saeki T, Kikuchi S (2012). The Effect of Controlling the Aging Condition on the Gelling Property of Silica Sols. Nihon Reoroji Gakkaishi.

[12] Kaide A, Saeki T (2014). Development of preparation method to control silica sol–gel synthesis with rheological and morphological measurements. Advanced Powder Technology.

[13] Schulz L (2011). Engineering spin propagation across a hybrid organic/inorganic interface using a polar layer. Nature materials.

[14] Chattopadhyay D, Zakula AD, Webster DC (2009). Organic–inorganic hybrid coatings prepared from glycidyl carbamate resin, 3-aminopropyl trimethoxy silane and tetraethoxyorthosilicate. Progress in Organic Coatings.

[15] Gizdavic-Nikolaidis MR, Zujovic ZD, Edmonds NR, Bolt CJ, Easteal AJ (2007). Spectroscopic characterization of GPTMS/DETA and GPTMS/EDA hybrid polymers. Journal of non-crystalline solids.

[16] Schmidt, H. & Krug, H. (ACS Publications, 1994).

[17] Metroke TL, Kachurina O, Knobbe ET (2002). Spectroscopic and corrosion resistance characterization of amine and super acid-cured hybrid organic–inorganic thin films on 2024-T3 aluminum alloy. Progress in organic coatings.

[18] Donley MS (2003). The self-assembled nanophase particle (SNAP) process: a nanoscience approach to coatings. Progress in organic coatings.

[19] Vargas MÁL, Montanari T, Delgado MCH, Alemany LJ (2005). Preparation and characterization of silicon hydride oxide: a fully hydrophobic solid. Journal of Materials Chemistry.

[20] Tiwari A, Zhu J, Hihara LH (2008). The development of low-temperature hardening silicone ceramer coatings for the corrosion protection of metals. Surface and Coatings Technology.

[21] Kupwade-Patil K, Palkovic SD, Bumajdad A, Soriano C, Büyüköztürk O (2018). Use of silica fume and natural volcanic ash as a replacement to Portland cement: Micro and pore structural investigation using NMR, XRD, FTIR and X-ray microtomography. Construction and Building Materials.

[22] Karakassides MA, Gournis D, Petridis D (1999). An infrared reflectance study of Si–O vibrations in thermally treated alkali-saturated montmorillonites. Clay Minerals.

[23] Yan Y, Hoshino Y, Duan Z, Chaudhuri SR, Sarkar A (1997). Design and Characterization of Interconnected, Microporous Hybrid Thin Films by a Sol− Gel Process. Chemistry of materials.

[24] Lin T-Y, Lee C-T (2012). Organosilicon function of gas barrier films purely deposited by inductively coupled plasma chemical vapor deposition system. Journal of Alloys and Compounds.

[25] Yu Y-Y, Chen C-Y, Chen W-C (2003). Synthesis and characterization of organic–inorganic hybrid thin films from poly (acrylic) and monodispersed colloidal silica. Polymer.

[26] Joseph R, Zhang S, Ford WT (1996). Structure and Dynamics of a Colloidal Silica− Poly (methyl methacrylate) Composite by 13C and 29Si MAS NMR Spectroscopy. Macromolecules.

[27] Chang T, Wang Y, Hong Y, Chiu Y (2000). Organic–inorganic hybrid materials. V. Dynamics and degradation of poly (methyl methacrylate) silica hybrids. Journal of Polymer Science Part A: Polymer Chemistry.

[28] Han Y-H, Taylor A, Mantle MD, Knowles KM (2007). UV curing of organic–inorganic hybrid coating materials. Journal of sol-gel science and technology.

[29] Bae WG (2012). One-step process for superhydrophobic metallic surfaces by wire electrical discharge machining. ACS applied materials & interfaces.

[30] Zhou J, Chen M, Qiao X, Wu L (2006). Facile Preparation Method of SiO2/PS/TiO2 Multilayer Core− Shell Hybrid Microspheres. Langmuir.

[31] De S, De G (2008). *In situ* generation of Au nanoparticles in UV-curable refractive index controlled SiO2− TiO2− PEO hybrid Films. The Journal of Physical Chemistry C.

[32] Tao P (2011). TiO 2 nanocomposites with high refractive index and transparency. Journal of Materials Chemistry.

[33] Nussbaumer RJ, Caseri WR, Smith P, Tervoort T (2003). Polymer‐TiO2 Nanocomposites: A Route Towards Visually Transparent Broadband UV Filters and High Refractive Index Materials. Macromolecular materials and engineering.

[34] Lü C, Guan C, Liu Y, Cheng Y, Yang B (2005). PbS/polymer nanocomposite optical materials with high refractive index. Chemistry of materials.

[35] Lü C, Cui Z, Li Z, Yang B, Shen J (2003). High refractive index thin films of ZnS/polythiourethane nanocomposites. Journal of Materials Chemistry.

[36] Chou T (2001). Organic–inorganic hybrid coatings for corrosion protection. Journal of Non-Crystalline Solids.

