# Supplementary material for: Functional Nano-Coating Materials by Michael Addition and Ring-opening Polymerization: Reactivity, Molecular Architecture and Refractive index
Source: Sci Rep. 2018 Aug 9;8:11912. doi: 10.1038/s41598-018-30458-x (PMC6085352; doi:10.1038/s41598-018-30458-x)
Supplement: Supplementary file 1 — Supplementary Information [file 41598_2018_30458_MOESM1_ESM.docx]

**Supplementary information**

**Functional Nano-Coating Materials by Michael Addition and Ring-opening Polymerization: Reactivity, Molecular Architecture and**

**Refractive index**

*Kishore K. Jena ^1, 2^, Saeed M. Alhassan ^2^, Atul Tiwari ^1^ and Lloyd H. Hihara ^1*^*

*^1^Hawaii Corrosion Laboratory (HCL)*

*Department of Mechanical Engineering*

*University of Hawaii at Manoa, Honolulu, HI 96822, USA*

*^2^Department of Chemical Engineering*

*Khalifa University of Science and Technology (KUST)*

*The Petroleum Institute, PO Box 2533, Abu Dhabi, United Arab Emirates (UAE)*


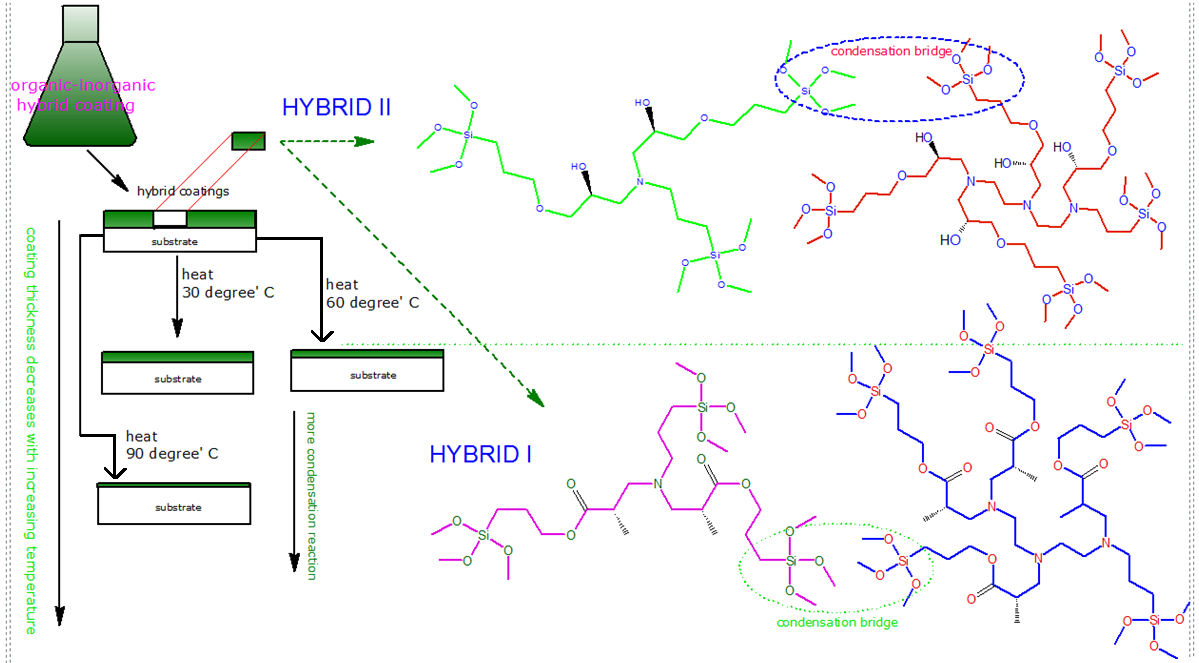


Scheme SI-1. Coating route and the Curing Process for Organic-Inorganic hybrid Networks. (HYBRID I and HYBRID II).


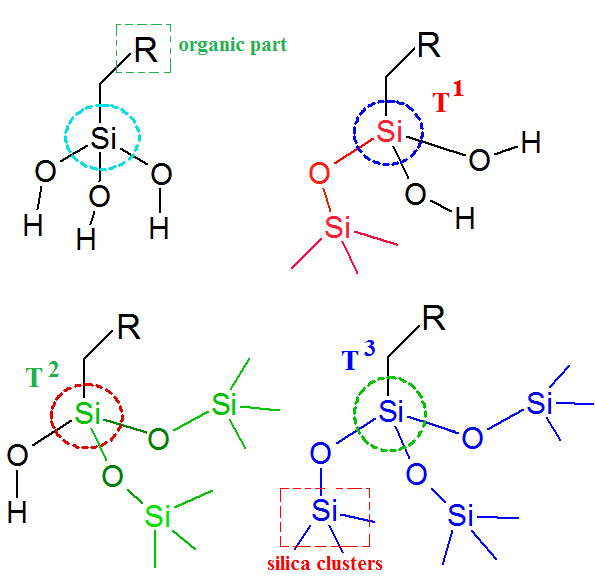


Scheme SI-2. Coating route and the Curing Process for Organic-Inorganic hybrid Networks. (HYBRID I and HYBRID II)


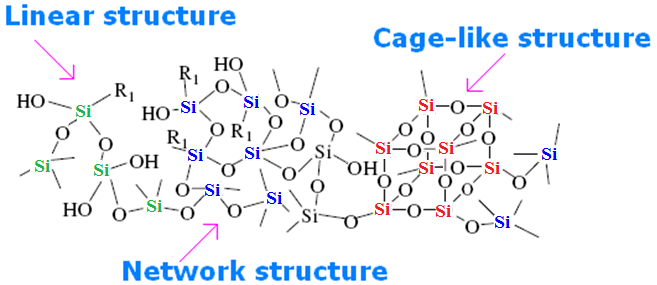


Scheme SI-3. Linear, cyclic (network) and branched (cage like) molecular structures.


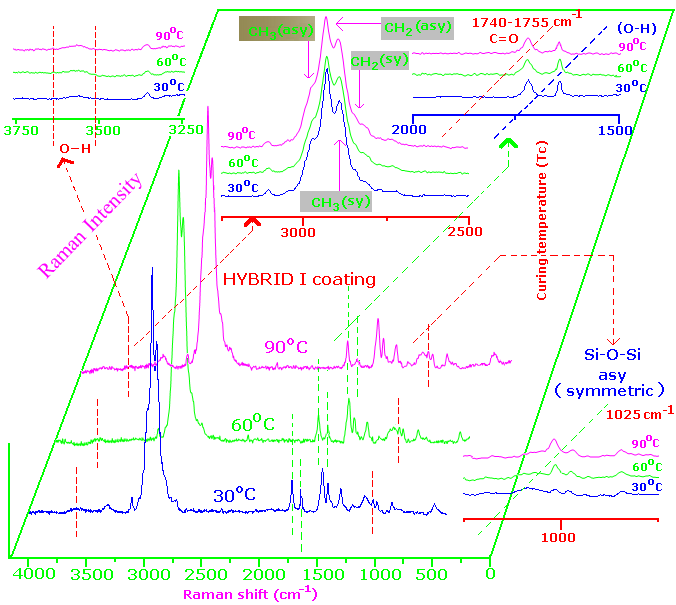


Figure SI-1. Full Raman Spectra of HYBRID I at different curing temperature (30, 60, and 90°C).


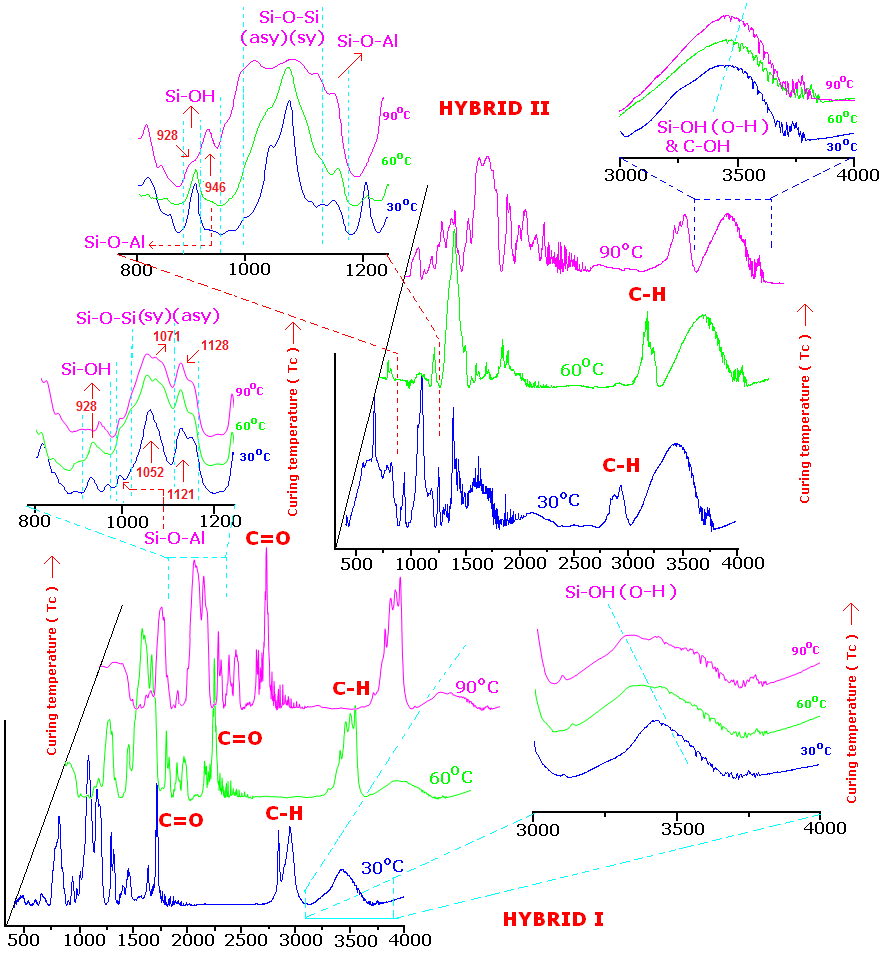


Figure SI-2. The FT-IR spectra of the organic-inorganic hybrids at different curing temperature (30, 60, and 90°C). The expanded zone of HYBRID I and HYBRID II in the spectral range 800-1200 and 3000-4000 cm^-1^.


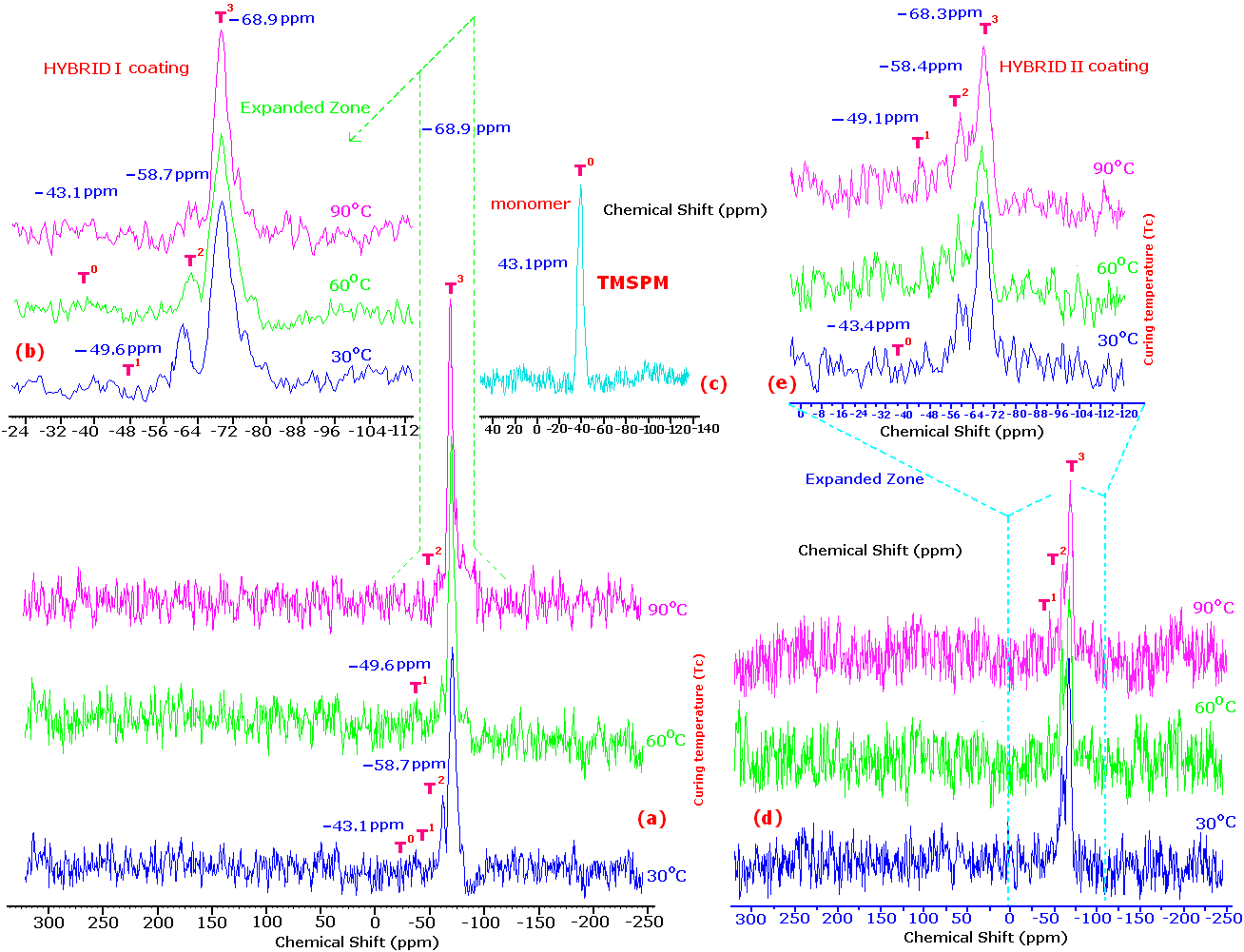


Figure SI-3. Solid state 29Si NMR spectra of cured hybrid materials. The expanded zone of hybrids in the ppm range -24 to -120 (shown above).

Table SI-1. The molar percentage of T species and degree of condensation (D_c_) for hybrid samples at different temperature.

| **HYBRID I** | | | | **HYBRID II** | | | |
| --- | --- | --- | --- | --- | --- | --- | --- |
| Molar percentage of T species (%) | | | | | | | |
| TEMP. | 30 | 60 | 90 | | 30 | 60 | 90 |
| T^1^ | 3.4 | 1.2 | 0.4 | | 5.3 | 1.6 | 0.6 |
| T^2^ | 33.9 | 32.4 | 28.8 | | 32.4 | 33.1 | 33.3 |
| T^3^ | 62.7 | 66.4 | 70.8 | | 62.3 | 65.3 | 66.1 |
| D_c_ | 86.4 | 88.4 | 90.1 | | 85.6 | 87.9 | 88.5 |

**Instrumental techniques**

The Raman spectra were obtained with a Thermoelectron Nicolet™ Almega™ XR dispersive Raman spectrometer equipped with an Atlus microscope and Omnic^®^ software. The 2024-T3 Al coupons were coated with the hybrid coatings and analyzed directly under the Atlus microscope. The conditions for the Raman Spectrometer and microscope system were set to the 532 nm laser, 80% laser power, 50 µm pinhole apertures, and low resolution. A background spectrum was collected prior to analyzing the sample.

The FTIR spectra were collected using a Thermo Electron Nicolet Nexus 760 instrument. The 2024-T3 Al coupons were coated with the hybrid coatings, and analyzed in reflectance mode. Spectra were obtained using 34 scans and a 4 cm^−1^ resolution. The spectra were analyzed using Thermo Electron's Omnic software. A blank background spectrum was collected prior to collecting a spectrum of the sample.

The inner structure of hybrid materials was studied using solid-state ^29^Si magic-angle spinning-nuclear magnetic resonance (MAS NMR). Measurements were performed on the hybrid coatings using a spectrometer (VARIAN Unity Inova) operating at 400 MHz.

The coating morphology and elemental analyses were conducted with a Hitachi S-4800 scanning electron microscope (SEM) and Oxford INCA Energy 250 energy-dispersive X-ray analyzer, respectively. The nanoscale structures of the silica clusters in the hybrid coatings were analyzed with a Hitachi HT7700 transmission electron microscope (TEM) operated at a 120 kV accelerating voltage. The specimens for the TEM observations were prepared by placing one drop of the coating precursor on a copper grid and heating at different curing temperatures. The refractive index of HYBRID I and HYBRDI II coatings were measured by variable angle spectroscopy Ellipsometer (VASE - 32, J.A. Woollam Co. Inc) in the wavelength range 400-1700 nm and angle of incidence 40 - 85° on a spin-coated sample on optical glass plate.

**Substrate Preparation and Film Deposition**

Aluminum 2024-T3 alloy was used as the substrate for the hybrid coatings. The composition of the AA 2024-T3 substrate was 93.3% Al, 0.5% Si, 0.5% Fe, 4.9% Cu, 0.3% Mn, 0.25% Zn, 0.15% Ti, and 0.10% Cr. The substrate was ground using 180-grit, 320-grit and 600-grit silicon carbide grinding paper on a Buehler Ecomet 6 variable speed grinder. After grinding with 600- grit paper, the alloy surface was rinsed with DI (deionized) water. The substrate was subsequently polished using 1 μm, 0.3 μm and 0.05 μm Buehler Micropolish II alumina suspensions. After polishing, the substrates surface were again rinsed with DI water and then cleaned with ethanol before air drying [1,2]. The polished aluminum alloy substrates were immersed into the hybrid sol for 5 min. The coating was air-dried onto the substrate and placed in an oven to cure at different temperatures (i.e., 30°C, 60°C, and 90°C) for 15 min to study the structure-morphology relationship of hybrid coatings. Metal specimens of 1×1 cm^2^ size were used for Raman, FTIR, and SEM experiments in this study.

**References**

1. Kasemann, R., Schmidt,H. Coatings for mechanical and chemical protection based on

organic-inorganic sol-gel composites. *New Jornal of Chemistry* **18** , 1117-1123(1994).

1. Tiwari, A., Hihara, L.H. High performance reaction-induced quasi-ceramic silicone

conversion coating for corrosion protection of aluminium alloys. *Progress in Organic Coatings*, **69**,16-25(2010).
